# Effect of Distillery Spent Wash Utilization on Maize Silage Fermentation and *In Vitro* Methane Production

**DOI:** 10.3390/ani15213146

**Published:** 2025-10-29

**Authors:** Yu Tang, Guangrou Lu, Hongxiang Zhao, Lin Li, Chaosheng Liao, Pan Wang, Yubo Zhang, Meiyan Zhang, Ping Li, Wenlong Gou

**Affiliations:** 1 Engineering Research Center of Biomass Materials, Ministry of Education, College of Life Sciences and Agri-Forestry, Southwest University of Science and Technology, Mianyang 621010, China; 2 College of Animal Science, Guizhou University, Guiyang 550025, China; 3 College of Animal Science and Veterinary Medicine, Southwest Minzu University, Chengdu 610041, China; 4 Grassland Animal Science Research Institute of Yunnan Province, Kunming 650220, China

**Keywords:** silage additives, silage storage, greenhouse gas, rumen fermentation, lactic acid bacteria

## Abstract

The animal husbandry industry is a major source of agricultural greenhouse gas emissions, and methods to reduce methane production from livestock during rumen fermentation are an important research issue. Distillery spent wash (DSW) is a by-product of the brewing industry, and to our knowledge, its application in feed processing has rarely been reported. This work investigated the effect of using DSW as an additive on silage fermentation and *in vitro* methane production. A small-scale silage production system for whole-plant maize was applied, and maize was treated without (control) and with DSW (G2, 10 mL·kg^−1^ fresh matter (FM); G4, 20 mL·kg^−1^ fresh matter). After 60 days of ensiling, the pH value of G2- and G4-treated whole-plant maize was reduced (*p* < 0.05), while lactic acid production was significantly enhanced to 3.40% DM for G2 and to 3.43% DM for G4, effectively improving silage fermentation. In fact, the addition of DSW shifted the bacterial community structure in whole-plant maize silage, significantly increasing the dominance of *Lactiplantibacillus* from 10.1% (CK) to 74.1% (G2) and 62.5% (G4) and thereby decreasing the diversity indices of silage samples. However, DSW application increased the ammonia-N levels in silage by 135.29–147.06% compared with the control. Importantly, there was a reduction of 0.70 and 0.55 percentage units in CK for G2 and G4, respectively, which represent 57.6% and 44.0% reductions in the acetic acid concentration, respectively, which were accompanied by a trend of decreasing methane production and *Methanobrevibacter* abundance. In conclusion, DSW exhibits potential for enhancing lactic acid fermentation by increasing *Lactiplantibacillus* and for inhibiting *in vitro* rumen methane production by promoting *Methanobrevibacter* dominance.

## Simple Summary

The animal husbandry industry produces large amounts of methane, a gas that contributes to global warming. Scientists are seeking practical methods to reduce the methane released during digestion in ruminants such as cattle. In this study, we explored whether a by-product of the alcohol industry—distillery spent wash—can be used as a natural additive in corn silage. We found that adding small amounts of this liquid promoted the growth of beneficial lactic acid bacteria, thereby improving fermentation quality while lowering the levels of undesirable acids. The treated silage also produced slightly less methane during simulated *in vitro* fermentation in the laboratory. This suggests that distillery spent wash could serve as a low-cost, environmentally friendly feed additive that both improves feed quality and helps reduce greenhouse gas emissions from livestock. At the same time, reusing this industrial by-product offers a sustainable way to reduce waste from the brewing industry.

## 1. Introduction

Greenhouse gases contribute to climate change on a global scale, with agricultural practices accounting for about 24 percent of global emissions [1]. Methane (CH_4_) is considered a powerful contributor to global warming and, together with carbon dioxide (CO_2_) and nitrous oxide (N_2_O), is one of the most influential greenhouse gases [2]. Methane emissions from ruminant animals are a significant agricultural pollutant and a major source of greenhouse gases in the atmosphere. Agriculture contributes 62% of global anthropogenic CH_4_ emissions, of which ruminants contribute 58% [3]. In addition, according to 2021 data from the Food and Agriculture Organization of the United Nations (FAOSTAT), CH_4_ emissions from livestock production account for 58.7% of the total agricultural emissions [4]. Ruminal microbial fermentation is the primary source of CH_4_ emissions in ruminants. Exploring the rumen microbial diversity and community structure, as well as the relationship between the efficiency of feed utilization and CH_4_ emissions, is crucial to reducing CH_4_ emissions [4]. Measures such as improving feed and applying additives have also proven effective to some extent in this regard [5]. When Wanapat M et al. added garlic to the diets of ruminants, it led to varying effects on CH_4_ emissions [6]. At high doses (300 mg/L), the *in vitro* effect was very significant (90% reduction), whereas the in vivo effect was less pronounced (for example, a 5% reduction was observed in beef cattle). The use of seaweed as a dietary additive has also been shown to have significant methane reduction potential [7].

Chemical and microbial additives are commonly applied to enhance silage quality and fermentation efficiency, improve feed nutritional value, and reduce *in vitro* rumen methane emissions by enhancing acid fermentation; however, large enterprises process large amounts of silage, and the additive costs increase in parallel with dosage. Secondly, with the addition of additives under ideal fermentation conditions, the improvement in quality may be less than the increase in cost, and thus the return on investment (ROI) is unclear, and there are diminishing benefits [8]. Therefore, it is necessary to develop new, low-cost, alternative additives. Zhang et al. used pomegranate peel, an agro-industrial by-product, as a silage additive to reduce *in vitro* rumen CH_4_ emissions and nitrogen losses (*p* < 0.05) [9], while Lu et al. reported the use of Yellow serofluid, a food industry by-product, as a silage additive for improving corn silage fermentation quality and reducing greenhouse gas emissions from rumen [10].

It has been shown that for every liter of alcohol produced, 8–15 L of DSW (a wine industry by-product) are generated, with the global annual alcohol production at more than 200 million tons [11]. DSW is a type of grain paste produced via distillation of water vapor in bad spirits, and it repeatedly condenses and sinks to the bottom of the pot of dark brown liquid. It has a high chemical oxygen demand (COD), low pH, and contains alcohols, esters, organic acids, sugars, vitamins, and other organic matter [12,13]. Improper disposal of DSW into open drains and landfills near distilleries leads to multiple environmental problems, for example, high nutrient levels can lead to eutrophication of water bodies, while colored water can block sunlight and inhibit photosynthesis, thus reducing oxidation and posing a threat to aquatic life [14]. Thus, the economic and effective handling of DSW has become a pressing problem in the liquor industry that must be solved for industry development. DSW has been employed in agroecosystems as a nutrient provider for plants. Field application, with high levels of N, P, and K at doses of 125 m^3^ ha^−1^ or lower, significantly improves crop growth and yield, whereas doses exceeding 250 m^3^ ha^−1^ may impair plant growth and adversely influence soil properties [15]. In addition, in countries such as India and Brazil, DSW has emerged as an inexpensive substitute for chemical fertilizers because it is readily available in large quantities [16]. However, DSW may have the potential for use in feed processing applications due to its organic acids, which can rapidly reduce the pH and inhibit clostridial fermentation, and residual sugars, which provide a fast-acting carbon substrate for lactic acid bacteria (LAB). Recent studies have explored the use of distillery wastewater (including DSW) as a *Sorghum propinquum* silage additive, showing that it significantly improved silage quality and aerobic stability [17].

This study aimed to evaluate the effects of DSW on fermentation and methane production in maize silage. It was hypothesized that application of DSW as an additive would enhance the fermentation quality of corn silage and affect the digestibility of ruminants, thus decreasing methane production. This would contribute to lowering greenhouse gas emissions from livestock while promoting the utilization of DSW.

## 2. Materials and Methods

### 2.1. Material Preparation

Maize plants were grown in Huaqiu Town, Zunyi City, Guizhou Province, China (28°7′ N, 106°34′ E). Whole plant maize at the milk stage was transported to the laboratory and cut into 2–3 cm sections and immediately subjected to treatment as follows: 5 mL·kg^−1^ of purified water (CK) on a fresh matter basis; 2% DSW(concentration), treated at a dose of 10 mL·kg^−1^ fresh matter (G2); and 4% DSW, treated at a dose of 20 mL·kg^−1^ fresh matter (G4). Additives were manually mixed with the chopped maize to ensure a uniform distribution. About 500 g of chopped materials was packed into polyethylene sachets (30 × 50 cm) and vacuum-sealed using a PW300 device (Qingye Packaging Machinery Co., Qingzhou, China). Each treatment included three independent silos (*n* = 3). After 60 days of ensiling at room temperature (25–30 °C), whole-plant maize silage was sampled to analyze the chemical and microbial compositions. Whole-plant corn (fresh material, FM) contained 27.97% dry matter (DM), 25.15% water-soluble carbohydrate (WSC) on a dry matter basis., with 9.51% crude protein (CP) (DM basis), 45.62% neutral detergent fiber (NDF) (DM basis), and 24.86% acid detergent fiber (ADF) (DM basis). The predominant epiphytic LAB present in pre-ensiled maize plants were *Lactobacillus acidophilus*, *L. cassava* and *L. panniculatus*.

### 2.2. Physical and Chemical Analyses

In short, a 20 g portion of each stored silage sample was added to 180 mL of sterile distilled water; after 3 min of uniform mixing with a laboratory juicer, the silage filtrate was filtered through four layers of sterile gauze. The sample solution pH was determined with a pH meter (PHS-3C, Yidian Scientific Instruments Co., Shanghai, China). About 20 mL of the filtrate was subjected to centrifugation (4500× *g*, 15 min, 4 °C). Ammoniacal nitrogen concentrations were measured according to the method of G.A. Broderick [18]. Lactic, acetic, propionic, and butyric acids in the supernatant were analyzed by high-performance liquid chromatography (HPLC; Thermo Scientific, Waltham, MA, USA) with a Shodex KC-811 column (8 mm × 300 mm; Shimadzu Co., Ltd., Tokyo, Japan) and a UV detector set at 210 nm [18]. The mobile phase consisted of 3 mmol/L perchloric acid, delivered at a flow rate of 1.0 mL/min, with the column temperature maintained at 50 °C and with an injection volume of 20 µL. Quantification was performed using external calibration with standard solutions of lactic, acetic, propionic, and butyric acids (Sigma-Aldrich, St. Louis, MO, USA) prepared in the range of 0.05–10 mmol/L (*R*^2^ > 0.999). The limits of detection (LOD) and quantification (LOQ) were 0.01 mmol/L and 0.03 mmol/L, respectively [18].

A total of 200 g of sample was weighed into an envelope and subsequently dried at 65 °C for over 60 h until a constant weight was reached, after which the dry matter (DM) content was determined. All results were corrected based on the laboratory dry matter percentage. The desiccated samples were ground and sieved through a 1 mm sieve and then sealed and stored in self-sealing bags for routine nutrient index determination. Neutral and acid detergent fibers were analyzed using the method described by Van et al. [19], and the total nitrogen content was determined using the Kjeldahl method with full automation; the CP content was calculated as TN × 6.25 [20]. The anthrone-sulfuric acid colorimetric method was used to determine the soluble carbohydrate content [21].

### 2.3. Bacterial and Archaeal Community Analysis

After 60 days of fermentation, biomass samples were collected and subjected to 16S rRNA gene sequencing analysis. Total genomic DNA was isolated from every stored sample using the CTAB method (cetyltrimethylammonium bromide method) and then stored at −80 °C for further analysis. The full-length 16S rRNA gene was amplified using barcoded, specific primers, with 806R and 515F employed for bacteria and 1837R and 1106F for archaea. Polymerase chain reaction (PCR) was carried out employing TransStart^®^ FastPfu DNA Polymerase (TransGen Biotech Co., Ltd., Beijing, China). Subsequently, the amplified 16S rRNA gene fragments were sequenced on the PacBio Sequel II platform. The raw sequencing data were processed by Novogene Bio-Technology Co., Ltd. (Beijing, China) using the QIIME2 pipeline (version 2023.2). Low-quality reads were filtered, and chimeric sequences were removed using the UCHIME (v4.2.40) algorithm. Amplicon sequence variants (ASVs) were generated with DADA2(v1.14.0), and taxonomic classification was performed based on the SILVA SSU rRNA database (release 138.1). To minimize sequencing depth bias, the ASV table was rarefied to the lowest sequencing depth across samples before downstream analyses. Functional inference and microbial community alpha diversity were further evaluated, and principal coordinate analysis (PCoA) was performed through the NovoMagic (version 3.0) platform. Alpha diversity indices—including Observed OTUs, Chao1, Shannon, Simpson, Pielou_e, Dominance, and Goods coverage—were calculated to assess bacterial community richness, evenness, and diversity using the vegan package in R (version 4.3.2). Beta diversity was assessed based on Bray–Curtis distances and visualized through principal coordinate analysis (PCoA). All raw sequence data were deposited in the NCBI Sequence Read Archive (SRA) under accession number PRJNA1301314.

### 2.4. In Vitro Ruminal Fermentation

This study was conducted in accordance with the UNIH Guide for the Care and Use of Laboratory Animals, complied with the Declaration of Helsinki, and was approved by the Institutional Review Committees for the Use of Human or Animal Subjects of Southwest University of Science and Technology and Guizhou University. Rumen fluid was collected from three freshly slaughtered yaks at a local abattoir. The bottom and middle portions of the rumen fluid were extracted with a vacuum suction device (to avoid sedimentation at the bottom) and immediately filtered through four layers of sterile gauze. The filtered rumen fluid was maintained at 39 °C under continuous CO_2_ flushing to preserve anaerobic conditions and was transported to the laboratory within 20 min in pre-warmed, insulated containers. The artificial rumen nutrient solution was prepared following the procedure of Menke et al. [22]. *In vitro* fermentation was carried out using a 250 mL fermenter. A fiber bag with a pore size of 25 μm (F57, ANKOM Technology, Macedon, NY, USA) was loaded with 1.5 g of sample (CK, G2, G4, FM) and quickly placed in the fermenter. A total of 200 mL of ruminal mixed fermentation broth was introduced into the fermentation flask with a measuring tube, and the experiment was conducted with the Animal Nutrient *In Vitro* Digestion Model System (Gas Endeavour III, BPC Instruments Co., Ltd., Haining, China).

The gas yield was measured at 3, 6, 9, 12, 24, 36, 48, 56, and 72 h following the initiation of fermentation, and automatically recorded by the Animal Nutrient *In Vitro* Digestion Model System. All gases were collected after 72 h, and the methane yield was analyzed using GC-2014 (Shimadzu, Kyoto, Japan) according to the method of Cai et al. [23]. The gas chromatography parameters and the calculation method for methane yield are consistent with the method of Cai et al. [23]. To evaluate the *in vitro* digestibility of dry matter (IVDMD), after 72 h of digestion, the residue was carefully washed with distilled water and subsequently dried at 65 °C for 48 h. At this stage, our aim was to assess the degradation characteristics of maize silage during the ruminal fermentation phase rather than to determine its total tract digestibility. Ammonia nitrogen concentrations were measured according to the method of G.A. Broderick [18]. Volatile fatty acids in the *in vitro* fermentation supernatant were analyzed using a gas chromatograph (Trace 1600, Thermo Scientific, Waltham, MA, USA) equipped with an AT-FFAP capillary column (30 m × 0.32 mm × 0.25 µm; Thermo Fisher Scientific, Waltham, MA, USA) and a flame ionization detector (FID). Nitrogen was used as the carrier gas at a constant flow rate of 1.0 mL/min, with a split ratio of 40:1 and an injection volume of 1 µL. The injector and detector temperatures were both set at 250 °C. The oven temperature was initially set at 90 °C, then increased at a rate of 20 °C per minute to 160 °C and held for 8.5 min, followed by a further rise of 10 °C per minute to 170 °C, where it was maintained for 2 min. The detection temperature was 250 °C [24]. To avoid confusion with silage acids, it should be noted that silage organic acids were determined by HPLC, while VFAs from *in vitro* fermentation were analyzed by GC–FFAP. Neutral and acidic detergent fibers were analyzed following the procedure of Van et al. [19]. The crude protein (CP) content in the residues was analyzed using the Kjeldahl method [20], and CP was calculated as total nitrogen (N) × 6.25. The values were calculated according to the following formulas: IVDMD = (DM content in the sample − undegraded DM)/DM content in the sample × 100%; IVCPD = (CP content in the sample − undegraded CP)/CP content in the sample × 100%.

IVNDFD = (NDF content in the sample− undegraded NDF)/NDF content in the sample × 100%; IVADFD = (ADF content in the sample − undegraded ADF)/ADF content in the sample × 100% [10].

### 2.5. Statistical Analysis

SPSS software 27.0.1 (IBM Corp., Armonk, NY, USA) was utilized for data analysis. A one-way analysis of variance (ANOVA) was used to evaluate the effects of the different treatments on the prepared silages (CK, G2, and G4). Fresh material (FM), although not silage, was used in the statistical analysis as a reference line for the analyses of bacterial population diversity and *in vitro* fermentation. Gas production was measured at multiple incubation times (3, 6, 9, 12, 24, 36, 48, 56, and 72 h), and these were analyzed separately using one-way ANOVA. The chemical composition and bacterial microbiota of silage were analyzed using Duncan’s multiple comparisons test. A Pearson’s correlation analysis was employed to analyze the association between fermentation parameters and the bacterial microbiota. Redundancy analysis (RDA) was performed in R software (version 4.3.2) using the vegan package to explore relationships between silage physicochemical parameters and bacterial community composition at the genus level. The analysis was based on Bray–Curtis distances and employed scaling type 2 (species-focused). Differences were regarded as statistically significant when the significance level was less than 0.05 (*p* < 0.05).

## 3. Results and Discussion

### 3.1. Chemical Composition of Silage Samples

The chemical composition of samples after 60 days of fermentation is shown in Table 1. From the table, it can be observed that after fermentation for 60 days, there were no significant differences in DM, CP, NDF, ADF, Ash and Hemicellulose (HC) between CK, G2 and G4 groups (*p* > 0.05). The loss in WSCs accompanying the silage process is mainly due to the metabolic consumption of these compounds by microorganisms in the silage system [25]. As expected, the results show that the WSC content of all groups after silage was lower than the WSC content of fresh corn (25.15% DM). Notably, in the CK treatment, WSC content was markedly higher than that of the G2 and G4 treatments (*p* < 0.05); in contrast, the pH was significantly higher compared with the G2 and G4 treatments (*p* < 0.05) (Table 2). This may be due to the fact that during CK fermentation, the growth rate of the raw material’s own microbial flora is slower than that of the exogenous LAB, and thus the WSCs are not consumed in a timely manner and acid production is insufficient, thus causing a slow decrease in pH. The higher lactic acid content in the G2 and G4 treatments also confirms this hypothesis.

### 3.2. Silage Fermentation Profile

The silage fermentation profile of samples at the end of the 60-day fermentation is shown in Table 2. The fermentation characteristics of silage are a key indicator of quality, with the pH serving as a direct sign of silage standard; high-quality silage typically has a pH below 4.2 [26]. Compared with the CK group, the pH values of G2 (4.03) and G4 (4.15) were markedly lower (*p* < 0.05). This may be due to the fact that G2 and G4 had notably higher lactic acid concentrations (3.40% and 3.43% DM). The lactic acid concentration of wine wastewater was as high as 970 mg/L in the experiments performed by Converti A et al. [27]. The lactate/acetate ratio was significantly higher for G2 (6.45) and G4 (4.93) than for CK (1.93) (*p* < 0.05). This was attributed to the dominance of homolactic LAB fermentation, in which water-soluble carbohydrates (WSCs) are efficiently converted into lactic acid with very little acetic acid by-product, resulting in a significantly lower acetic acid concentration compared with CK (*p* < 0.05). In addition, the G2 (0.70% DM) and G4 (0.68% DM) treatments exhibited the greatest propionic acid content, which was notably higher than that of the CK group. Propionic acid, owing to its antimicrobial activity, is commonly applied to delay heating in silage and enhance aerobic stability in open air. It also partially suppresses Clostridia and decreases the formation of butyric acid (an undesirable product), which explains the relatively low butyric acid content observed in the G2 and G4 treatments [28]. Notably, the highest ammonia-N contents were observed in G2 (0.93% DM) and G4 (1.03% DM), both of which were notably greater than that of the CK group. This may be due to the higher ammonia-N concentration in DSW; Sarayu Mohana et al. found a total of 5000–7000 mg/L of total nitrogen in DSW [29]. In summary, we conclude that the silage quality was indeed improved following fermentation with DSW relative to the CK group. The use of DSW also promotes waste reutilization and offers potential economic and environmental benefits compared with commercial additives, providing valuable evidence supporting the utilization of DSW.

### 3.3. Analysis of Bacterial Communities in Whole-Plant Maize Silage Samples

Table 3 illustrates the bacterial α-diversities, a general indicator of the richness of a species community within a single sample [30], of the samples. From Table 3, it was concluded that DSW treatment significantly reduced species richness in silage (Chao 1). G2 and G4 treatments differed from the control in all indices, showing significantly lower Shannon and Simpson values (*p* < 0.05). This reduction in diversity may be attributed to the selective enrichment of certain dominant bacterial taxa, such as *Lactiplantibacillus*, during the ensiling process. Ren et al. found that LAB inoculation reduced the Shannon and Simpson indexes of the bacterial community in alfalfa, suggesting a similar trend in which the microbial community may have become more specialized under DSW treatment [31].

**Figure 1:**
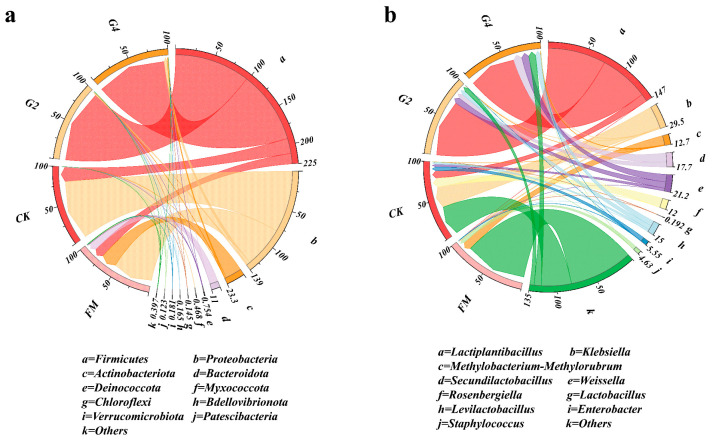
Relative abundances of dominant phylum (**a**) and genera (**b**). FM, fresh materials; CK, without additives; G2, inoculation with 2% DSW; G4, inoculation with 4% DSW.

Figure 1a,b present the relative abundances of the dominant phylum and genera in the silage samples, respectively. During the silage process, microorganisms (e.g., Lactobacillus, *Bacillus*) interact with each other. The dominant phylum of FM is *Proteobacteria*, followed by *Actinobacteria*. The CK, G2, and G4 treatments exhibited an increased relative abundance of *Firmicutes* compared with FM. The relative abundance of *Firmicutes* in the fermented samples was usually significantly increased compared with fresh materials, which was mainly attributed to the proliferative advantage of *Lactobacillus* under this phylum in anaerobic environments [32]. The dominant phylum *Firmicutes* accounted for more than 90% of G2 and G4 treatments, thus showing that DSW increased the proportion of *Firmicutes*. At the genus level, *Klebsiellas* represented the dominant taxon in CK and YS. *Klebsiella* belongs to the *Enterobacteriaceae* family and is an acid-tolerant parthenogenetic anaerobe that can flourish under anaerobic conditions by absorbing fermentation substrates, and competition for fermentation substrates by *Klebsiella* may account for the lower abundance of *Lactiplantibacillus* [33]. Compared to CK, fermentation increased the abundance of *Weissella*, an obligate heterofermentative lactic acid bacterium that favors silage fermentation by metabolizing carbohydrates to produce lactic acid to lower the pH. G2 and G4 treatments resulted in an increase in the abundance of *Lactiplantibacillus*, which became the predominant genus, and a decrease in the abundance of *Klebsiella*, which explains the higher concentration of lactic acid in G2 and G4 than in CK. It is noteworthy that the abundance of *Lactiplantibacillus* accounted for 74% in the G2 treatment, which explains the lower Shannon and Simpson indices in the G2 treatment. In summary, the addition of DSW improved the fermentation quality.

**Figure 2:**
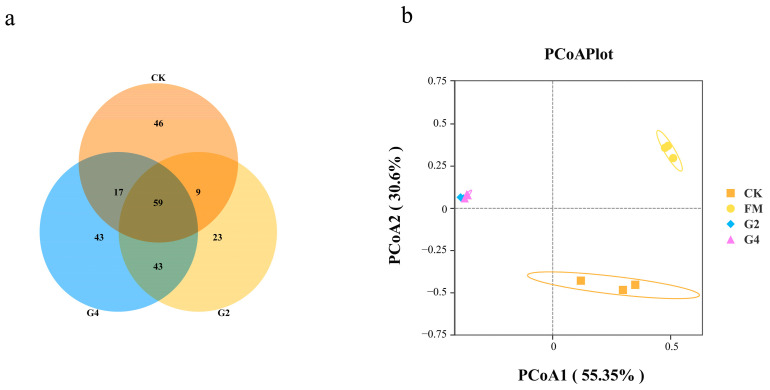
Venn diagram (**a**) and PCoA plot of the bacterial community structure (**b**) of silage samples. FM, fresh materials; CK, without additives; G2, inoculation with 2% DSW; G4, inoculation with 4% DSW.

Figure 2 shows a Venn diagram (a) and PCoA plot (b) of the silage bacterial community. A total of 59 shared OTUs constituted the core bacterial community of the silage samples. The exclusive OTUs identified per treatment ranged between 23 in G2 and 46 in CK. The addition of 2% DSW reduced bacterial diversity. The contribution of PCoA1 and PCoA2 to the total variance was 48.21% and 35.33%, respectively. The G2 and G4 treatments had the shortest distances, followed by the FM and CK treatments, and clustered together in quadrant II, while the CK treatment was located in quadrant IV. Silage samples with similar bacterial microbiota structures showed a tendency to cluster together, while shifts in bacterial community structure revealed differences in silage quality [34]. Therefore, G2 and G4 treatments exhibited the most similar bacterial community structure, with the addition of DSW causing changes in this structure in whole-plant corn silage.

### 3.4. Correlation Analysis Between Bacterial Community and Environmental Conditions of Samples After 60 Days of Fermentation

**Figure 3:**
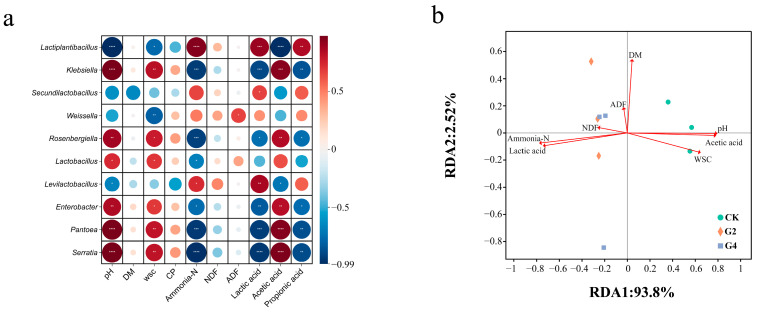
Correlation analysis of environmental factors with genus-level bacteria (**a**). The color gradient indicates the Pearson correlation coefficients of the sample taxa after 60 days of fermentation. * Significant at *p* < 0.05; ** *p* < 0.01; *** *p* < 0.001; **** *p* < 0.0001. Redundancy analysis (RDA) of correlations between microbial communities based on genus-level bacteria and physicochemical parameters (**b**). CK, without additives; G2, inoculation with 2% DSW; G4, inoculation with 4% DSW; WSC, water-soluble carbohydrate; DM, dry matter; CP, crude protein; NDF, neutral detergent fiber; ADF, acid detergent fiber.

The correlation analysis between environmental factors and genus-level bacteria is shown in Figure 3a, revealing the association between environmental factors and bacterial communities in silage. The main roles of the LAB in silage are to convert WSCs to lactic acid, lower pH and promote fermentation [35]. In this study, *Lactiplantibacillus* was significantly negatively correlated with pH and acetic acid, but positively correlated with lactic acid, suggesting that *Lactiplantibacillus* is the main producer of lactic acid during fermentation. On the other hand, this genus was dominant in the DSW treatments, which explains the higher lactic acid content in the G2 and G4 treatments, along with the lower acetic acid content. *Klebsiella* can inhibit lactic acid fermentation by converting amino acids to toxic nitrogen oxides and, secondarily, by consuming soluble sugars in silage to compete with *Lactiplantibacillus* for resources [36]. In the present study, *Klebsiella* (which poses a risk to animal health) showed a notable positive association with pH and acetic acid and a negative association with lactic acid, which, as the dominant genus of CK, further proves that the fermentation quality of the CK treatment was lower than that of the DSW treatment. Notably, *Lactiplantibacillus* showed a significant negative correlation with ammonia-N, suggesting that *Lactiplantibacillus* may degrade this compound.

Redundancy analysis (RDA) of the correlations between physicochemical properties and microbial populations on the basis of genus-level bacterial communities is shown in Figure 3b. RDA allows for visualization of the association between bacterial communities and environmental parameters and for a better understanding of the variation in silage quality across treatment groups. The RDA results showed that the correlations between environmental parameters and bacterial communities showed substantial variation across treatments. The bacterial communities of G2 and G4 treatments differed from those of CK, being positively associated with lactic acid and ammonia-N while negatively associated with pH, acetic acid, and WSCs. The addition of DSW led to the dominance of lactic acid fermentation in the G2 and G4 treatments, which is consistent with the fact that *Lactiplantibacillus* was the dominant genus in the DSW treatments. Elevated ammonia-N in conventional silage usually predicts spoilage (excessive breakdown of proteins by *Clostridium* spp.), but the G2 and G4 treatments were significantly enriched in lactic acid along with ammonia-N; thus, their ammonia-N content may be directly derived from DSW [37]. Overall, the correlation analysis between bacterial communities and environmental factors confirmed that the application of DSW facilitated lactic fermentation in silage.

### 3.5. Parameters of In Vitro Fermentation and Methane Emissions

We conducted the *in vitro* fermentation experiments based on the silage experiments. The *in vitro* fermentation parameters are shown in Table 4. Ruminal pH is a key indicator of the stability of the rumen’s internal environment [38], with, overall, a low pH markedly suppressing the activity and functionality of rumen microorganisms and thereby influencing the hydrolysis, acidification, and methanogenesis stages of the rumen digestive process [39]. The pH of all treatments was within the normal range, but there was a significant difference (*p* < 0.05) in pH between treatments, with that of the G2 group being significantly higher than the other groups. Y. Meng et al. reported that a neutral pH (6.9) during rumen fermentation was more conducive to corn stover hydrolysis and CH_4_ production [40]. In contrast, the pH of the G4 and FM treatments was closer to 6.9, suggesting that there may be higher methane production in these two groups. The ammonia-N produced during rumen fermentation is an important nitrogen source for rumen microorganisms, supporting their growth and proliferation. However, the accumulation of ammonia-N in the G2 and G4 treatments also suggests that the efficiency of assimilation of nitrogen into microbial protein was relatively low, implying that, despite the increased nitrogen supply, the potential for microbial protein synthesis was reduced, probably due to the WSC concentration (4.41 and 4.32% versus 5.72% for CK). Microbial growth depends on the ratio of readily available nitrogen and carbohydrate sources.

VFAs are methanogenic intermediates produced during the acidification stage of anaerobic organic matter fermentation [41]. Acetic acid was the main component in this experiment, followed by propionic and butyric acids, which is in agreement with most studies [42]. The G4 treatment had the highest concentration of acetic acid (57.12 mmol/L), which was significantly higher than that of the FM and G2 treatments (*p* < 0.05), but was not significantly different from CK. While the accumulation of large amounts of acetic acid facilitates methane production during subsequent anaerobic fermentation, VFAs other than acetic acid cannot be directly utilized by methanogenic bacteria [43]. The next highest concentrations of propionic, butyric, and valeric acids were found in the FM and G4 treatments, and were not significantly different. Propionic and butyric acids are first converted to acetic acid, CO_2_ and H_2_ by acetic acid-producing bacteria and then utilized by acetic acid-producing methanogens and hydrogenotrophic methanogens. Suyun Xu et al. found that the methanogenic capacity was slightly inhibited in biogas digesters supplemented with propionic acid because the metabolism of propionic acid prevented the methanogenic bacteria from producing methane [44]. In addition, according to Calabrò et al., the production of acetate and butyrate is linked with the release of CO_2_ from both microbial metabolism and the reaction of acid with bicarbonate buffer [45]. Isovaleric acid was markedly higher in the G4 treatment than in G2 (*p* < 0.05). Few investigations have addressed the oxidative decomposition of isovaleric acid, with Stieb et al. indicating that acetic acid and H_2_ are generated during its degradation, but that this process does not proceed through the β-oxidation pathway [46].

**Figure 4:**
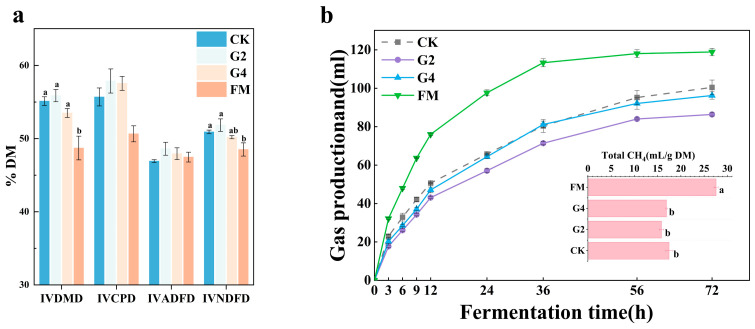
*In vitro* digestibility (**a**) and total gas and methane production (**b**) from *in vitro* digestion of silage samples. FM, fresh materials; CK, without additives; G2, inoculation with 2% DSW; G4, inoculation with 4% DSW. IVDMD, *In Vitro* Dry Matter Digestibility; IVCPD, *In Vitro* Crude Protein Digestibility; IVADFD, *In Vitro* Acid Detergent Fiber Digestibility; IVNDFD, *In Vitro* Neutral Detergent Fiber Digestibility. Differences between means with different superscripts (a,b) are significant (*p* < 0.05). No letter indicates no significant difference.

The VFAs produced by rumen microbes during *in vitro* fermentation should be stoichiometrically related to gas production [47]. The total gas produced and the methane yield from *in vitro* digestion of whole-plant corn silage samples are shown in Figure 4b. The total gas production of FM was notably higher than that of the silage treatments, while methane production was also significantly higher (*p* < 0.05). This result agrees with the study of D.W. et al., who found that rumen-fermented methane production was higher in fresh sugar beets than in post-silage beets [48]. The lowest total gas production was observed for the G2 treatment. Although methane production did not differ significantly from that of CK (*p* > 0.05), a slight decreasing trend was observed (15.83 vs. 17.46 mL/g DM). We suggest that the lower concentration of acetic acid in silage in the G2 treatment was responsible for this. In addition, although the G4 treatment had the highest concentration of acetic acid (which is favorable for the methanogenic process), its propionic and butyric acid contents were also relatively high, so methane production was not significantly higher (16.9 mL/g DM), as expected. The G2 treatment had the lowest methane production, so we conclude that the use of 2% DSW as a silage additive may have the potential to inhibit methane production during rumen fermentation, which could be advantageous for reducing greenhouse gas emissions generated by livestock.

The *in vitro* digestibility of whole-plant maize silage is displayed in Figure 4a. From this figure, it was observed that the *in vitro* digestibility of the post-silage samples was significantly improved compared to that of fresh whole-plant maize, in agreement with the study of Niu et al. [30]. The higher IVDMD of the post-silage samples was responsible for the increases in neutral detergent solute and cellulose digestibility. Increasing the IVDMD promotes rumen microbial fermentation and improves the nutrient utilization rate in feed [49]. It is remarkable that IVCPD was quantitatively significantly lower in FM than in the other groups, yet there was no significant difference. The higher IVCPD values in G2 and G4 may be due to the higher abundance of *Lactiplantibacillus*, which breaks down large protein molecules into more easily digestible forms. The G2 group exhibited higher *in vitro* rumen digestibility than the other groups (but these differences were only significant in the FM group), potentially enhancing the overall digestive efficiency of the rumen.

### 3.6. Analysis of Archaea Community and Functional Relative Abundance of Silage

**Figure 5:**
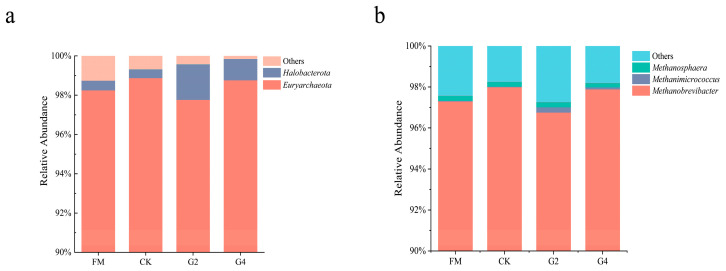
Relative abundance of the dominant phylum (**a**) and genera (**b**) of archaea in *in vitro* Fermentation. FM, fresh material; CK, without additives; G2, inoculation with 2% DSW; G4, inoculation with 4% DSW.

**Figure 6:**
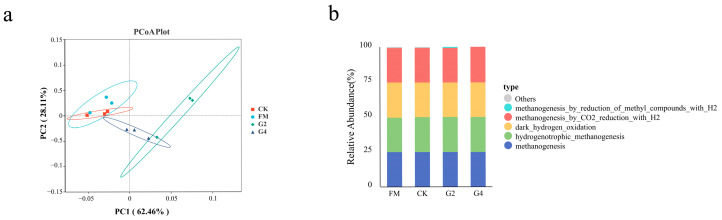
PCoA plot of the archaea community structure (**a**) and functional relative abundance based on FAPROTAX analysis (**b**) *in vitro* fermentation. FM, fresh material; CK, without additives; G2, inoculation with 2% DSW; G4, inoculation with 4% DSW.

Table 5 presents the α-diversities of Archaea in the silage samples. The coverage index reached 1.00 in all samples, implying that most archaeal taxa were identified through this sequencing approach. Of the archaeal diversity indices assessed, there were marked differences (*p* < 0.05) between the three index groups of Pielou-e, Shannon and Simpson. DSW treatment significantly reduced species richness (Chao 1), but maintained a high species diversity (Shannon), evenness (Pielou-e), and dominance (Simpson) in *in vitro* fermentation, suggesting that the addition of DSW suppressed the activity of certain microorganisms while retaining highly competitive dominant bacteria and maintaining an even species distribution. Figure 5a,b present the distribution of archaeal relative abundance at the phylum and genus levels during *in vitro* fermentation. *Euryarchaeota*, identified as the major phylum for all treatments, is a critical microbial group in anaerobic digestion, encompassing several methanogenic taxa (such as *Methylobacillus* and *Methanobacterium*) responsible for converting intermediates to methane in anaerobic environments [50]. It is noteworthy that the G2 treatment had a lower abundance of the *Euryarchaeota* phylum (97.8%) compared to the other treatments. On the genus scale, *Methanobrevibacter* (strictly dependent on H_2_ and CO_2_ or formic acid for methanogenesis) was the dominant genus in all treatments but had the lowest abundance (96.7%) in G2, and *Methanobrevibacter*, *Methanimicrococcus*, *Methanosphaera* and *Methanocorpusculum* also had the lowest total abundance (97.2%) in the G2 treatment (compared to 97.6%, 98.3% and 98.2% in the FM, CK and G4 treatments, respectively). This accounts for the lower methane production in the G2 treatment. The PCoA of the Archaea community structure in the *in vitro* fermentation broth of silage corn is shown in Figure 6a. PCoA1 and PCoA2 contributed 62.46% and 28.11%, respectively, to the total variance. The CK and FM treatments had the shortest distances between samples, while the G2 treatment had the longest. The PCoA plots illustrate that DSW-treated silage did not have the same archaeal community structure as those of FM and CK in the *in vitro* fermentation environment; thus, the DSW treatment induced changes in the archaeal community structure in *in vitro* fermentation. In summary, we hypothesize that G2 treatment inhibits the activity of *Methanobrevibacter* spp. However, specific reasons for this (e.g., changes in substrate availability or inhibition by environmental stress) were not thoroughly investigated in our experiments.

The functional relative abundance in the *in vitro* fermentation broth of silage corn is shown in Figure 6b, based on FAPROTAX analysis. Methanogenesis, hydrogenotrophic_methanogenesismethanogenesis_by_CO_2__reduction_with_H_2_ and m-ethanogenesis_by_reduction_of_methyl_compounds_with_H_2_ are all key metabolic functions predicted by FAPROTAX analysis to be associated with the methanogenic process, and it can be observed that the relative abundance of the above metabolic functions in the G2 and G4 treatments was not notably different from that of CK, suggesting that the potential of these specific methanogenic functions did not appear to be significantly elevated as a result of the addition of DSW. Thus in *in vitro* fermentation, DSW-treated silage does not show a higher functional potential for methanogenesis, but changes in the abundance of key genera (e.g., reduced abundance of *Methanobrevibacter*) potentially reduce methanogenesis via the substrate competition pathway.

## 4. Conclusions

Using DSW as a silage additive can effectively reduce the pH and improve the silage quality by inhibiting the growth of stray bacteria. The addition of 2% DSW to corn silage reduces the acetic acid concentration by 57.6%, with a tendency toward a reduction in methane production due to the abundance of the *Methanobrevibacter* population. These findings indicate that DSW has the potential to be used as a silage additive, and provide new insights into the valorization of DSW.

## Figures and Tables

**Table 1:** Chemical composition of whole-plant maize after 60 days of fermentation.

Items	CK	G2	G4	SEM	*p*-Value
DM, % FM	32.57	32.78	32.19	0.264	0.764
CP, % DM	10.11	9.73	9.92	0.122	0.503
WSC, % DM	5.72 ^a^	4.41 ^b^	4.32 ^b^	0.721	0.001
NDF, % DM	61.95	62.60	62.22	0.251	0.751
ADF, % DM	36.00	35.72	36.20	0.184	0.707
Ash, %	2.15	2.15	2.07	0.027	0.461
HC, %	25.95	26.87	26.02	0.062	0.383

CK, without additives; G2, inoculation with 2% DSW; G4, inoculation with 4% DSW; SEM, standard error of the mean; DM, dry matter; CP, crude protein; NDF, neutral detergent fiber; ADF, acid detergent fiber; HC, hemicellulose. ^a,b^ indicate significant differences between values in the same row at *p* < 0.05.

**Table 2:** Fermentation characteristics of whole-plant maize after 60 days of fermentation.

Items	CK	G2	G4	SEM	*p*-Value
pH	4.80 ^a^	4.03 ^c^	4.15 ^b^	0.357	<0.001
Lactic acid, % DM	2.35 ^b^	3.40 ^a^	3.43 ^a^	0.603	0.012
Acetic acid, % DM	1.25 ^a^	0.53 ^b^	0.70 ^b^	0.338	<0.001
Lactic acid/Acetic acid	1.93 ^c^	6.45 ^a^	4.93 ^b^	0.686	<0.001
Propionic acid, % DM	0.63 ^b^	0.70 ^a^	0.68 ^a^	0.039	0.021
Butyric acid, % DM	0.36	0.16	0.20	0.103	0.053
Ammonia-N, % DM	0.34 ^b^	0.80 ^a^	0.84 ^a^	0.251	<0.001

CK, without additives; G2, inoculation with 2% DSW; G4, inoculation with 4% DSW; SEM, standard error of the mean. ^a–c^ indicate significant differences between values in the same row at *p* < 0.05.

**Table 3:** Bacterial α-diversity in samples after 60 days of fermentation and fresh materials.

Items	FM	CK	G2	G4	SEM	*p*-Value
Goods coverage	0.861 ^b^	0.972 ^a^	0.985 ^a^	0.978 ^a^	0.016	<0.001
Observed Otus	527.33 ^a^	145.33 ^b^	60.00 ^c^	87.67 ^c^	198.320	<0.001
Dominance	0.01 ^c^	0.06 ^c^	0.41 ^a^	0.32 ^b^	0.177	<0.001
Chao1	844.43 ^a^	204.93 ^b^	93.74 ^c^	135.34 ^bc^	93.158	<0.001
Pielou_e	0.87 ^a^	0.73 ^b^	0.40 ^c^	0.44 ^c^	0.059	<0.001
Shannon	7.88 ^a^	5.20 ^b^	2.38 ^c^	2.84 ^c^	0.662	<0.001
Simpson	0.99 ^a^	0.94 ^a^	0.59 ^c^	0.68 ^b^	0.051	<0.001

FM, fresh material; CK, without additives; G2, inoculation with 2% DSW; G4, inoculation with 4% DSW; SEM, standard error of the mean. ^a–c^ indicate significant differences between values in the same row at *p* < 0.05.

**Table 4:** *In vitro* fermentation parameters of whole-plant maize silage and fresh material.

Items	CK	G2	G4	FM	SEM	*p*-Value
pH	6.85 ^c^	7.11 ^a^	6.96 ^b^	6.92 ^b^	0.031	<0.001
NH_3_-N (mmol/L)	8.33 ^a^	9.19 ^a^	8.70 ^a^	7.38 ^b^	1.436	0.008
TVFAs (mmol/L)	77.55 ^b^	77.18 ^b^	84.25 ^a^	76.44 ^b^	1.088	0.008
Acetate (mmol/L)	54.57 ^ab^	52.89 ^b^	57.12 ^a^	48.11 ^c^	1.069	<0.001
Propionate (mmol/L)	12.54 ^c^	13.82 ^bc^	15.19 ^ab^	16.07 ^a^	0.445	0.002
Butyrate (mmol/L)	6.39 ^b^	6.49 ^b^	7.44 ^a^	8.00 ^a^	0.232	0.007
Isobutyrate (mmol/L)	1.15 ^ab^	1.11 ^ab^	1.25 ^a^	1.07 ^b^	0.028	0.129
Valerate (mmol/L)	1.01 ^c^	1.04 ^bc^	1.16 ^ab^	1.17 ^a^	0.026	0.037
Isovalerate (mmol/L)	1.89 ^ab^	1.83 ^b^	2.10 ^a^	2.01 ^ab^	0.041	0.080

FM, fresh materials; CK, without additives; G2, inoculation with 2% DSW; G4, inoculation with 4% DSW; SEM, standard error of the mean. ^a–c^ indicate significant differences between values in the same row at *p* < 0.05.

**Table 5:** Archaea α-diversity of samples in *in vitro* fermentation.

Item	FM	CK	G2	G4	SEM	*p*-Value
Goods coverage	1.00	1.00	1.00	1.00	-	-
Observed Otus	121.67 ^a^	108.00 ^ab^	88.67 ^ab^	69.67 ^b^	28.970	0.117
Dominance	0.82 ^a^	0.82 ^a^	0.67 ^c^	0.75 ^b^	0.065	<0.001
Chao1	127.00 ^a^	118.46 ^ab^	90.12 ^ab^	69.85 ^b^	9.452	0.097
Pielou_e	0.13 ^c^	0.13 ^c^	0.22 ^a^	0.19 ^b^	0.012	<0.001
Shannon	0.89 ^c^	0.90 ^c^	1.42 ^a^	1.15 ^b^	0.069	<0.001
Simpson	0.18 ^c^	0.18 ^c^	0.33 ^a^	0.25 ^b^	0.019	<0.001

FM, fresh materials; CK, without additives; G2, inoculation with 2% DSW; G4, inoculation with 4% DSW; SEM, standard error of mean. ^a–c^ indicate significant differences between values in the same row at *p* < 0.05.

## Data Availability

The data presented in the study are deposited in the NCBI repository (https://www.ncbi.nlm.nih.gov/ (accessed on 4 August 2025)), accession number PRJNA1301314.
